# Unusual fetal ascites and spontaneous bladder rupture in a female fetus: a case report

**DOI:** 10.1186/s13256-020-02425-6

**Published:** 2020-07-19

**Authors:** Florence Cadoret, Edith Brazet, Agnès Sartor, Isabelle Lacroix, Charlotte Casper, Stéphane Decramer, Olivier Parant

**Affiliations:** 1 CHU Toulouse, Pole de Gynécologie Obstétrique, Hôpital Paule de Viguier, 31059 Toulouse, France; 2 Centre Régional de Pharmacovigilance Midi-Pyrénées, 31000 Toulouse, France; 3 CHU Toulouse, Hôpital des Enfants, 31059 Toulouse, France; 4 Université de Toulouse III, UMR1027, 31073 Toulouse, France; 5 Inserm UMR1027, 31073 Toulouse, France

**Keywords:** Fetal bladder rupture, Prenatal uroperitoneum, Morphine derivatives, Fetal ascites, Prenatal diagnosis

## Abstract

**Background:**

Fetal bladder rupture causing urinary ascites is uncommon. It is generally related to invasive fetal medicine procedures or obstructive disorders such as in posterior urethral valves in male fetuses. An exceptional case of spontaneous bladder rupture in a female fetus occurred in a pregnant woman treated with high doses of opiates in an intensive care unit. This unusual obstetric situation leads to discussion of the possible causes of fetal bladder rupture, its management, and the pediatric prognosis.

**Case presentation:**

We report the case of a 30-year-old nulliparous black woman with a history of mesenteric cystic lymphangioma and multiple bowel resections leading to chronic malabsorption. During her pregnancy, our patient presented with an occlusive syndrome and major bilateral renal dilation. Urinary derivation resulted in iatrogenic bilateral ureteral perforation. Our patient thus presented with major uroperitoneum, bilateral pleural effusion and acute renal failure, treated by thoracic drainage and bilateral nephrostomy. Postoperative pain required treatment with level III analgesics. In this context, 5 days after morphine treatment introduction an enlarged fetal bladder was observed, followed 3 days later by voluminous fetal ascites. The diagnosis of spontaneous bladder rupture was suspected. After multidisciplinary discussion, expectant management was decided. At 31 weeks and 4 days gestation, our patient went into spontaneous labor with a subsequent vaginal delivery. The infant required resuscitation and paracentesis of ascites at birth. Her neonatal course was favorable with a simple urethral bladder drainage. Cystography at day 9 was normal. At 2 years of follow-up, the mother and the child have a normal course.

**Conclusions:**

An iatrogenic origin of megacystis in a female fetus must be evoked in the event of maternal administration of high doses of opiates in the second part of her pregnancy. In our case, the megacystis was followed by spontaneous bladder rupture at 30 weeks of gestation, with a favorable maternal fetal issue.

## Introduction

Fetal bladder rupture resulting in urinary ascites is a rare occurrence. It is most often linked to invasive fetal medicine procedures or obstructive pathologies, such as in posterior urethral valves in male fetuses [1]. We report an exceptional case of spontaneous bladder rupture in a female fetus at 30 weeks of gestation (WG) in a patient hospitalized in the intensive care unit (ICU) for iatrogenic uroperitoneum. This isolated obstetric case opens up the discussion to possible etiologies of fetal bladder rupture, management methods and the pediatric prognosis.

## Case presentation

We report the case of a 30-year-old nulliparous black woman with a history of primary mesenteric cystic lymphangioma requiring multiple bowel resections in childhood. This history was responsible for chronic malabsorption resulting from short bowel syndrome, for which our patient received long-term monthly supplemental parenteral nutrition. She weighed 41 kg for 1.57 m at the beginning of pregnancy (a body mass index [BMI] of 16.6).

Her first trimester of pregnancy followed a normal course and combined aneuploidy screening did not identify increased risk (combined risk 1/10,000). A control ultrasound at 22 WG showed a eutrophic female fetus with no observable morphological abnormalities (particularly of the urinary tract and abdomen) or excess amniotic fluid.

At 26 WG, our patient presented with mild to moderate bowel occlusion on computed tomography (CT) scan. Medical treatment involving nasogastric intubation and parenteral nutrition supplementation provided rapid though transient clinical improvement. After multidisciplinary consultation, it was decided to maintain exclusive parenteral nutrition until the end of the pregnancy due to signs of recurrent occlusion at 28 WG.

At 29 WG, our patient was hospitalized for bilateral lumbar pain. A renal ultrasound revealed dilatation of the bilateral pyelocaliceal cavities (27 mm on the right, 30 mm on the left) attributed to compression by the gravid uterus. Given the persistent pain and a slight rise in serum creatinine (63 to 84 micromol/L), an internal ureteral bypass with double J stents was rapidly performed. The procedure proved to be difficult due to ureteral siphons hindering the insertion of the stents and resulted in accidental bilateral ureteral perforation. Postoperative pain required the use of level III analgesics: oral morphine: immediate release oxycodone 5 mg 4 times a day and nefopam 120 mg continuous intravenous daily.

At 29 weeks and 4 days gestation, our patient was transferred to an ICU near the level 3 maternity ward. A CT scan revealed significant uroperitoneum and bilateral pleural effusion. Worsening of respiratory manifestations and onset of acute renal impairment (serum creatinine 180 micromol/L, potassium 5.2 mmol/L) required thoracic drainage (1900 mL) and bilateral nephrostomies. Our patient’s nephrological and respiratory condition rapidly improved.

During her ICU stay, our patient presented painful uterine contractions, for which she received tocolytic treatment with atosiban. For the persistent low back pain, morphine was continued intravenously at a mean dose of 50 mg of oxynorm per day (patient-controlled analgesia). Antenatal corticosteroid therapy was given. The fetal heart rate (FHR) was normal.

**Fig. 1:**
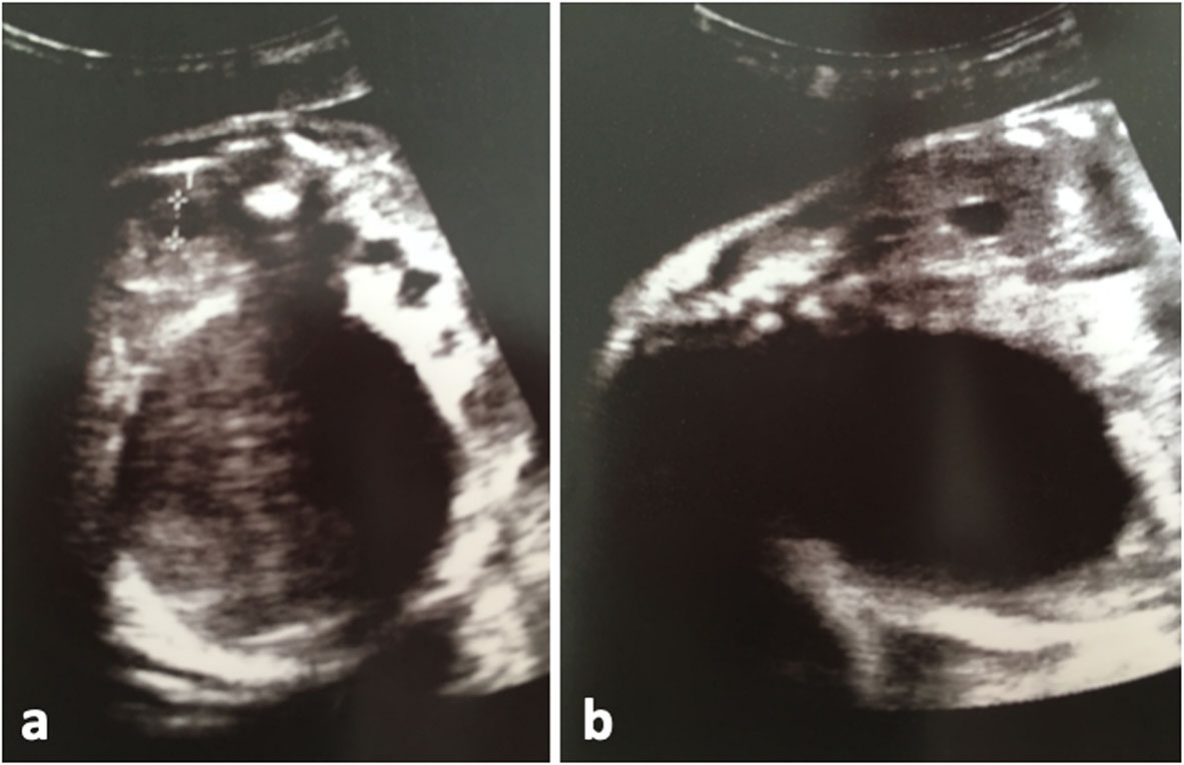
Fetal ultrasound at 28 weeks and 5 days. **a** Cross-section and (**b**) longitudinal: megacystis with renal impact. Presence of bilateral pyelocaliceal dilatation

On ultrasound, the fetus was eutrophic (estimated fetal weight [EFW]: 1250 g at 29 weeks and 3 days), in cephalic presentation, with a moderate excess of amniotic fluid (cisterna magna 112 mm) and increased bladder volume. At 29 weeks and 5 days gestation, 5 days after parenteral introduction of high-dose opioids, fetal megacystis increased (50 mm × 52 mm) and was associated with pyelocaliceal dilatation (Fig. 1).

**Fig. 2:**
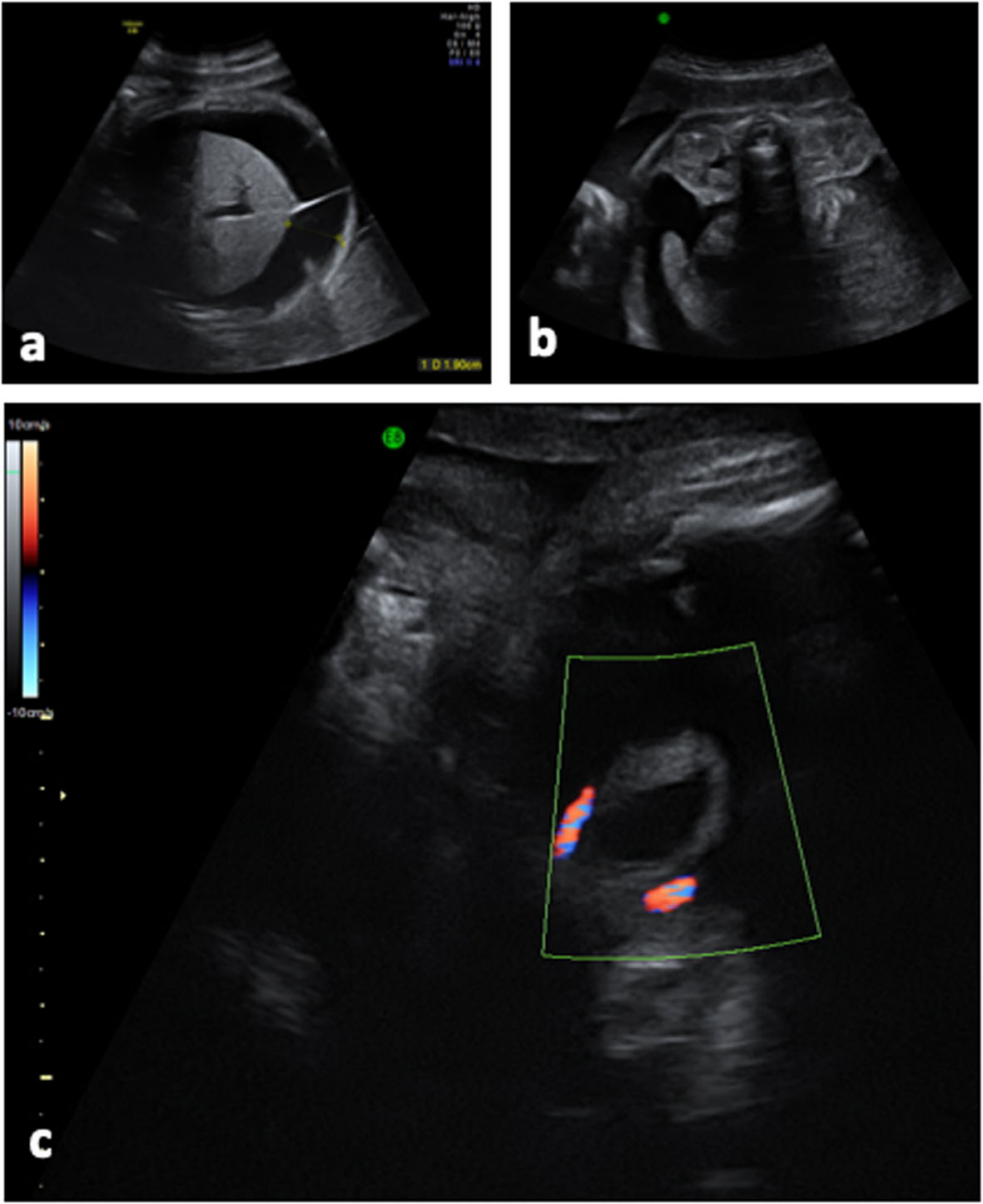
Fetal ultrasound at 30 weeks and 1 day. (**a**) Fetal ascites, (**b**) fetal ascites, normal appearance of the kidneys, (**c**) small capacity bladder with thickened walls

Three days later, an ultrasound revealed voluminous fetal ascites and a small bladder with thickened walls (3 mm) (Fig. 2). The fetal kidneys were morphologically normal. No anasarca or sign of fetal anemia (normal middle cerebral artery peak systolic velocity) was evident and the amniotic fluid index was normal. Possible spontaneous bladder rupture with urinary peritonitis was suggested. In the absence of a clear cause, the morphine derivatives administered to the mother, for 8 days (from 29 to 30 + 1 WG), were suggested as a contributing factor to the fetal megacystis.

After multidisciplinary discussion (pediatric surgeon, pediatric nephrologist, and obstetrician), anticipatory management was decided. Urgent extraction or draining of antenatal ascites were not performed because fetal hemodynamics were satisfactory. Our patient was closely monitored with three fetal heart rate (FHR) recordings per day and two ultrasounds per week. The maternal and fetal condition remained stable for 9 days (from 30 weeks and 1 day to 31 weeks and 3 days) and the fetal bladder remained unchanged throughout the monitoring.

At 31 weeks and 4 days gestation, our patient went into labor spontaneously and received epidural anesthesia. Labor was rapid and marked by moderate FHR abnormalities. Our patient gave birth vaginally to a girl weighing 1700 g, presenting moderate ischemic anoxia with Apgar scores at 1/1/10 and umbilical cord pH 7.19 (artery) and 7.27 (vein). Neonatal intensive care included mask ventilation followed by orotracheal intubation and external cardiac massage. Paracentesis of ascites (300 mL citrinic fluid) was carried out at 8 minutes. The infant was then transferred to the ICU. Her respiratory state required tracheal instillation of surfactant with a favorable respiratory outcome. Her hemodynamic condition was stable. No definite neonatal infection was identified, and intravenous antibiotic treatment was maintained for 8 days with cefotaxim based on the notion of peripheral ampicillin-resistant *Escherichia coli* carriage. Enteral feeding was initiated on the first day with the mother’s milk and then with specific infant formula for preterm neonates, followed by central line parenteral nutrition for 15 days. Transfontanellar cranial ultrasounds showed a grade 1 intraventricular hemorrhage with no parenchymal lesion.

Ascites drained at birth showed a biochemical profile similar to plasma, in favor of uroperitoneum. No metabolic disorder was found except for a plasma creatinine level at 79 μl/L on day 1 which quickly normalized within the first week of life. A urinary catheter was inserted following delivery. The abdominal ultrasounds performed on day 1 and day 6 were normal with a normal bladder wall. Cystography was normal on day 9 and the bladder catheter was removed. Ascites did not recur. The infant’s course was favorable without complications during hospitalization.

Regular pediatric monitoring was performed after discharge and showed a strictly normal outcome at last follow-up at 2 years of age (adjusted). The mother has been regularly monitored and has presented no complication. From a medical standpoint, a new pregnancy is not recommended.

## Discussion

The main causes of megacystis in a female fetus are related to an obstruction or stenosis of the lower urinary tract, bladder diverticula obstructing the bladder outlet, a neuropathy or a myopathy [2]. However, often no cause can be found. In the male fetus, the most common cause is posterior urethral valves. In our case, a possible iatrogenic origin linked to maternal repeated opiates administration (morphine, oxycodone, nefopam) during eight consecutive days was suggested. It may be assumed that the exposed fetus developed *in utero* megacystis, followed by spontaneous rupture, subsequent to the administration of morphine to her mother. The chronology of this pharmacologically plausible, potential undesirable effect is compatible. This clinical case has been comprehensively studied by the regional pharmacovigilance center of the university hospital. The intrinsic imputability of the onset of urinary complications to morphine and its derivatives was classified as I2 (on a scale ranging from I0 “excluded” to I4 “very likely”). The case has been entered in the national pharmacovigilance database.

Opiates may cause urinary retention by direct action on the bladder and by central inhibitory action on micturition [3]. In addition, these effects may be increased because of the immaturity of muscle and interstitial tissue.

There have been previously published cases of significant urinary dilatation leading to bladder rupture with uroperitoneum. A similar case of megacystis with bladder rupture was reported in a fetus whose mother was treated with midazolam and morphine between 32 and 33 WG. Rupture of the bladder associated with abundant uroperitoneum was observed at 34 WG during an ultrasound for decreased active fetal movements. The infant was delivered by cesarean section with a favorable pediatric outcome after 1 month of urinary drainage [4]. In addition, Bengtsson reported two cases of bladder distension with hydronephrosis in preterm neonates who received morphine [3].

In the case we report, the normal cystography on day 1 of life did not exclude this diagnosis since the bladder rupture would have taken place approximately 9 days before birth. In addition, the biochemical composition of the ascitic fluid and absence of recurrence support this hypothesis [5].

Fetal uroperitoneum has a biochemical composition similar to plasma, where the peritoneum acts as a filter [6], and it is not toxic to the peritoneum or bowel loops. The impact of fetal uroperitoneum is difficult to assess in terms of pain. The severity of fetal manifestations is more often related to chronic diaphragmatic compression resulting in pulmonary hypoplasia whose neonatal impact is difficult to predict [7].

There are no recommendations as to antenatal management of suspected uroperitoneum. Several therapeutic options are available to obstetric teams: fetal extraction in the short term, installation of a bypass system (peritoneal-amniotic shunt), or expectant management. The main risk linked to *in utero* insertion of a ureteral bypass system is preterm delivery, which is more often the result of premature rupture of the membranes (risk 5–7%). There are no current data related to peritoneal-amniotic shunt because it is extremely rare. However, the incidence of premature rupture of membranes is probably similar to that observed in vesicoamniotic shunts [6, 8]. Other complications have also been reported, such as displacement of the bypass system, oligoamnios or hydramnios, sepsis and laparoschisis [6]. Dysfunctions or complications from a ureteral bypass system range between 45% and 60% [8]. The benefit/risk ratio must be carefully considered before resorting to this type of therapy.

The therapeutic choice must be guided by gestational age, volume of uroperitoneum and its impact (particularly hemodynamic), maternal condition, and the couple’s wishes. In our case, due to the high degree of prematurity (uroperitoneum discovered at 30 WG) and absence of major impact on the fetus, after the couple had been fully informed a wait-and-see attitude seemed to be the most appropriate solution.

Neonatal management consisted of initial peritoneal drainage of ascites and permanent insertion of a bladder stent. Performing paracentesis of ascites from the time of birth onward seems to be useful in improving the respiratory state and facilitating resuscitation [9]. Bladder perforation can be treated either by a permanent bladder stent, leaving the bladder at rest for healing, or surgically if the perforation is large [10]. In all cases, cystography and/or cystoscopy for monitoring should be performed during days 10–14 and before removing the stent to check that the bladder is completely healed [10].

## Conclusions

The main causes of megacystis in a female fetus are obstruction of the lower urinary tract, neuropathy or myopathy. As in our case, an iatrogenic origin of megacystosis in a female fetus should be considered when high doses of opiates are administered to the mother in the second half of pregnancy. Spontaneous bladder rupture is one of the possible complications when bladder distension becomes significant. In the event of fetal bladder rupture, several therapeutic options are available: short-term fetal extraction, placement of a peritoneo-amniotic shunt, or expectant management. The therapeutic choice must be guided by gestational age, volume of uroperitoneum and its impact (especially hemodynamic), maternal condition, and the couple’s decision. As in our case report, expectant management may be a relevant option in the absence of major impact on the fetus and a high degree of prematurity.

## Data Availability

The patient’s file is available on demand.

## Abbreviations

BMI: Body mass index
CT: Computed tomography
EFW: Estimated fetal weight
FHR: Fetal heart rate
ICU: Intensive care unit
WG: Weeks of gestation
